# Pheochromocytoma Presenting as Spontaneous Adrenal Hemorrhage in a Normotensive Patient: A Case Report

**DOI:** 10.3390/jcm15186938

**Published:** 2026-09-08

**Authors:** Jokūbas Ramanauskas, Vestina Strakšytė, Inga Zaborienė, Kristina Žvinienė, Milda Jasevičė

**Affiliations:** 1 Medical Academy, Lithuanian University of Health Sciences, 44307 Kaunas, Lithuania; 2 Department of Radiology, Medical Academy, Lithuanian University of Health Sciences, 44307 Kaunas, Lithuania

**Keywords:** pheochromocytoma, adrenal hemorrhage, normotension, computed tomography, adrenalectomy

## Abstract

**Background**: Pheochromocytoma is a rare tumor of the adrenal medulla, often associated with hypertension, headache, diaphoresis, and palpitations, but presentations vary; some are silent until incidentally found or when complications develop. One rare complication is spontaneous adrenal hemorrhage, previously mainly found at autopsy, which can extend into the retroperitoneum, causing acute pain and mimicking emergencies. Imaging modalities like computed tomography are vital for detection, especially in acute cases, while magnetic resonance imaging provides greater tissue detail to identify tumors such as pheochromocytomas. If a functional adrenal tumor is suspected, biochemical tests for catecholamine excess are crucial for diagnosis and management. In this report, we present a case of pheochromocytoma that initially manifested with spontaneous adrenal hemorrhage without hypertension, thereby complicating and prolonging the diagnostic process. **Case presentation**: A previously healthy 34-year-old man presented with sudden, severe right-sided abdominal and flank pain, nausea, vomiting, and headache. He was normotensive but anemic and required a red blood cell transfusion. Initial computed tomography revealed a large hemorrhagic lesion in the right retroperitoneal space. The hematoma was managed conservatively. Magnetic resonance imaging confirmed subacute hematoma. Follow-up computed tomography showed gradual regression of the hematoma but persistence of an enhancing right adrenal lesion. Elevated plasma metanephrine and normetanephrine levels, together with positive iodine-123 metaiodobenzylguanidine imaging and preoperative computed tomography, supported the diagnosis of pheochromocytoma. After preoperative alpha-adrenergic blockade, laparoscopic right adrenalectomy was performed without complications. Histopathology confirmed a pheochromocytoma classified as moderately differentiated (GAPP), with a Ki-67index of 1–3%. Genetic testing showed no pathogenic variants in the analyzed genes associated with hereditary pheochromocytoma or paraganglioma syndromes. **Conclusions**: Spontaneous adrenal hemorrhage may be the first sign of pheochromocytoma, even in normotensive patients. Acute bleeding can obscure the tumor. Persistent adrenal lesions after hematoma regression should be monitored with computed tomography or magnetic resonance imaging, along with biochemical and functional evaluation, to avoid serious complications or death.

## 1. Introduction

Pheochromocytoma is an uncommon neuroendocrine tumor characterized by the secretion of catecholamines, originating from chromaffin cells within the adrenal medulla [1]. The incidence has increased from approximately 0.2 cases per 100,000 persons per year in studies performed before 2000 to approximately 0.6 cases per 100,000 persons per year in studies conducted after 2010, likely reflecting a shift toward more frequent detection through incidental imaging findings rather than diagnosis at autopsy or based on symptoms [1].

Clinically, pheochromocytoma is classically associated with paroxysmal or sustained hypertension, headache, diaphoresis, and palpitations. However, its presentation is often heterogeneous, and some tumors remain clinically silent until detected incidentally or after the development of complications [2,3].

One of its complications, spontaneous adrenal hemorrhage, is rare and underrecognized. Before the widespread use of computed tomography (CT), adrenal hemorrhage was mainly reported as an autopsy finding, with an incidence of 0.14–1.8% in large unselected postmortem series [4]. It can result from trauma, extreme physical stress, or the presence of pre-existing adrenal pathology [4]. Among tumors, pheochromocytoma is notable, often bleeding more than other adrenal tumors and accounting for nearly half of tumor-related hemorrhages [4,5]. This highlights the importance of diagnosis, as tumor rupture or intratumoral bleeding increases perioperative risk and mortality, especially if not identified before surgery [5]. Adrenal hemorrhage may remain limited within the tumor or may extend into the retroperitoneal space, producing acute abdominal or flank pain and mimicking other surgical emergencies [4,5]. Radiological imaging plays a central role in the evaluation of adrenal hemorrhage and suspected adrenal tumors [4]. CT is usually the first-line imaging modality in acute presentations, allowing rapid detection of adrenal masses and retroperitoneal bleeding [5]. In contrast, magnetic resonance imaging (MRI) provides additional tissue characterization and may help identify an underlying tumor such as pheochromocytoma [4,5]. Once a functional adrenal tumor is suspected, biochemical testing for catecholamine excess is essential for confirming the diagnosis and guiding further management [2].

Recognizing hemorrhagic pheochromocytoma is crucial because its management substantially differs from that of non-functioning adrenal hemorrhage. Confirming catecholamine secretion and starting preoperative alpha-adrenergic blockade are essential, as patients may face hypertensive crises or arrhythmias during surgery [2,6]. In cases where imaging shows a mass, or there are triggers such as a history of symptoms or palpitations, pheochromocytoma should be suspected [4,5].

In this report, we present a case of pheochromocytoma that initially manifested with spontaneous adrenal hemorrhage without hypertension, thereby complicating and prolonging the diagnostic process.

## 2. Case Presentation

**Figure 1:**
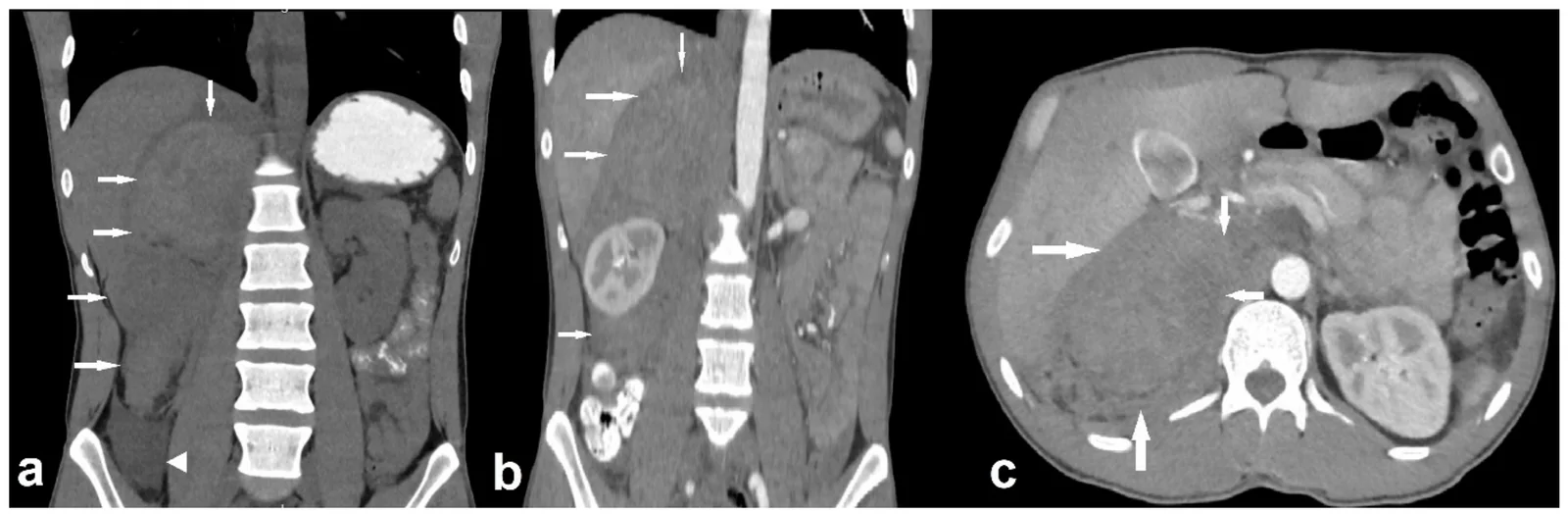
Initial CT images: (**a**) a coronal view without contrast showing a high-density hematoma (white arrows) in the right retroperitoneal space; (**b**) a coronal arterial phase image; and (**c**) an axial arterial phase image, all showing no signs of active bleeding (arrows). Additionally, free liquid is observed in the retroperitoneal space (arrowhead).

A 34-year-old man with no prior health issues or medication usage in December 2023 experienced a sudden onset of severe abdominal pain, primarily localized to the right flank and epigastric region, accompanied by nausea, vomiting, and headache. On admission, he was hemodynamically stable, with a blood pressure of 117/85 mmHg and a heart rate of 74 beats/min. The patient had no history of hypertension, and repeated blood pressure measurements during evaluations and at home remained normal, with no hypertensive episodes recorded. Laboratory investigations revealed anemia with a hemoglobin level of 93 g/L, and the patient subsequently required red blood cell transfusion. Due to acute abdominal pain and a decline in hemoglobin concentration, an urgent contrast-enhanced abdominal CT was performed. CT scan (Figure 1) revealed a large, irregular mass adjacent to the right adrenal gland, measuring approximately 8.9 × 7.8 cm. The lesion demonstrated mixed density, with surrounding blood extending into adjacent tissues, without active bleeding. The patient was hospitalized and managed conservatively with intravenous fluid therapy and blood transfusion due to decreased hemoglobin levels. After clinical stabilization, the patient was discharged with a scheduled follow-up for further diagnostic evaluation.

**Figure 2:**
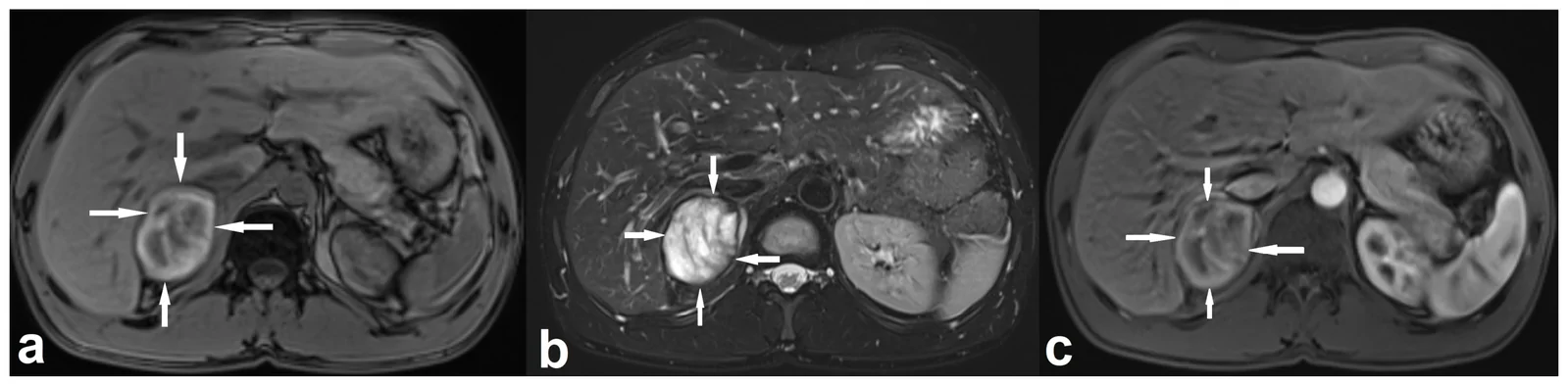
Axial MRI images: (**a**) T1-weighted pre-contrast image, (**b**) fat-suppressed T2-weighted image, and (**c**) T1-weighted post-contrast arterial-phase image demonstrating the heterogeneous right adrenal hemorrhagic lesion (arrows).

To better characterize the lesion, an MRI was performed in February 2024 (Figure 2). The scan revealed a right adrenal mass measuring approximately 5.5 × 4.0 cm with heterogeneous signal intensity and areas of central cystic degeneration. According to the radiological interpretation, the lesion was most consistent with a subacute hematoma in the right adrenal region.

**Figure 3:**
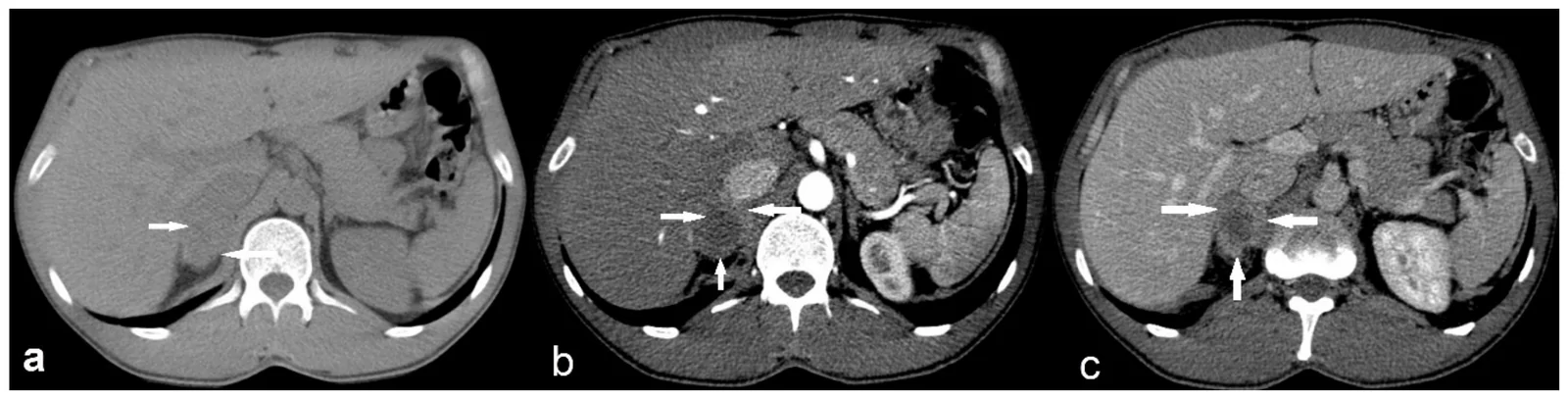
First follow-up CT axial images: (**a**) pre-contrast, (**b**) arterial phase, (**c**) venous phase showing the adrenal lesion decreased in size, with central hypodensity and peripheral contrast enhancement (arrows).

A follow-up CT scan conducted in June 2024 demonstrated a persistent right adrenal lesion that had decreased in size, measuring approximately 3.3 × 3.0 cm, with central hypodensity and peripheral contrast enhancement (Figure 3). Based on these findings, a pheochromocytoma was suspected, and the patient was referred for endocrinological evaluation. Plasma free metanephrines were measured using an enzyme immunoassay. Blood samples were obtained under standardized preanalytical conditions, with the patient in the supine position after 20–30 min of rest. The patient was not taking any medications at the time of testing. Initial biochemical evaluation in November 2024 demonstrated modest elevations of plasma free metanephrine (0.79 nmol/L; upper reference limit 0.507 nmol/L) and normetanephrine (1.91 nmol/L; upper reference limit 1.18 nmol/L). Repeat measurements in January 2025 (metanephrine 0.78 nmol/L, normetanephrine 2.47 nmol/L) and September 2025 (metanephrine 0.99 nmol/L, normetanephrine 3.83 nmol/L) confirmed persistent, progressively increasing elevations. Chromogranin A was only minimally elevated at the initial evaluation (104.67 µg/L; reference range 0–100 µg/L) and subsequently increased to 133.42 µg/L. Given its limited specificity, this finding was considered supportive rather than diagnostic.

**Figure 4:**
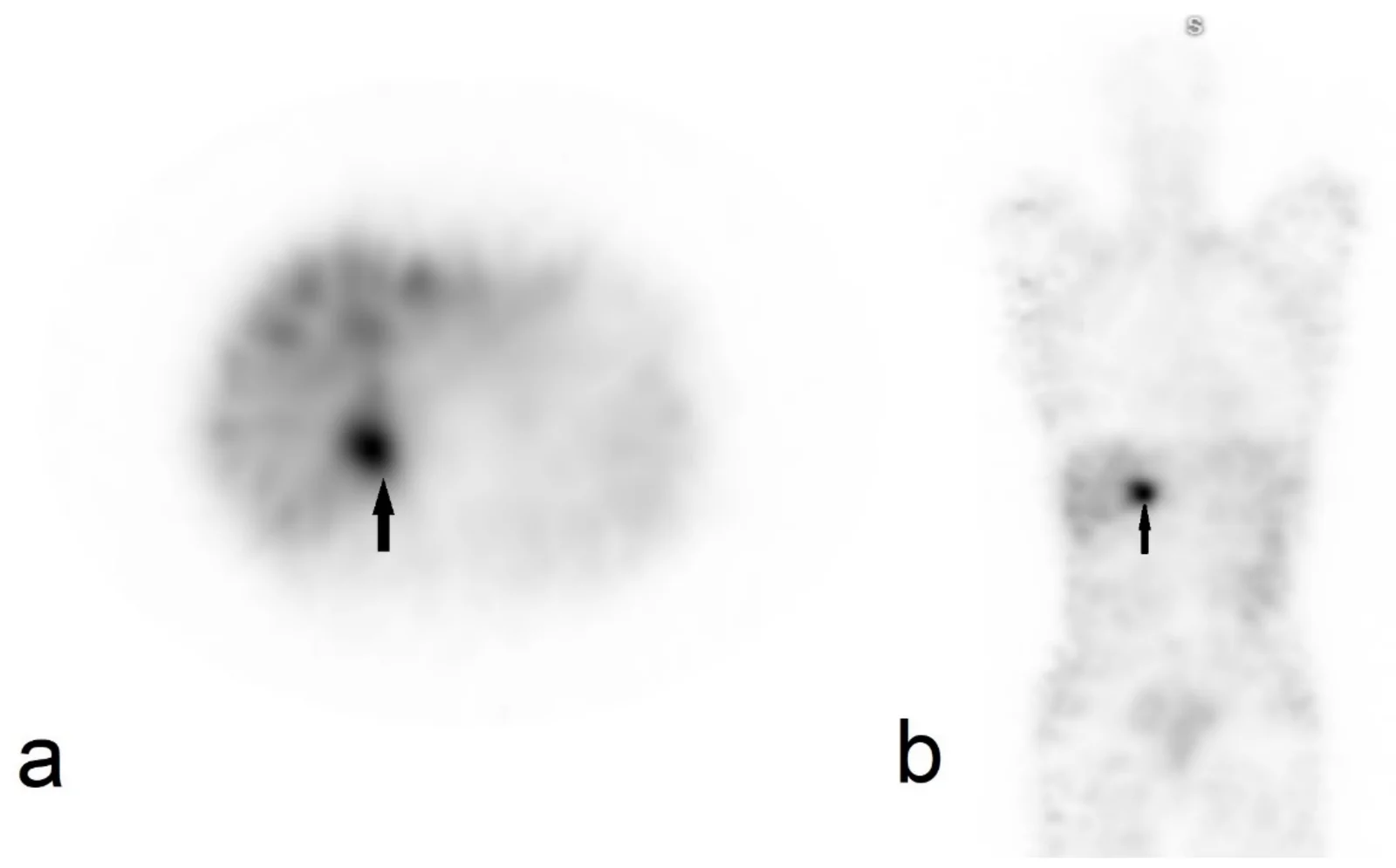
^123^I-MIBG imaging shows (**a**) axial and (**b**) coronal views, with notable ^123^I-MIBG uptake in the right adrenal gland (arrows).

For functional characterization of the lesion, iodine-123 metaiodobenzylguanidine (^123^I-MIBG) single-photon emission computed tomography (SPECT)/CT was performed in March 2025 (Figure 4). The examination demonstrated pathological radiotracer uptake localized to the right adrenal gland, consistent with an adrenergic tumor, while no additional pathological uptake suggestive of metastatic disease was detected. This modality was selected to confirm the functional, adrenergic nature of the persistent adrenal lesion and to screen for occult metastatic disease before surgical planning.

**Figure 5:**
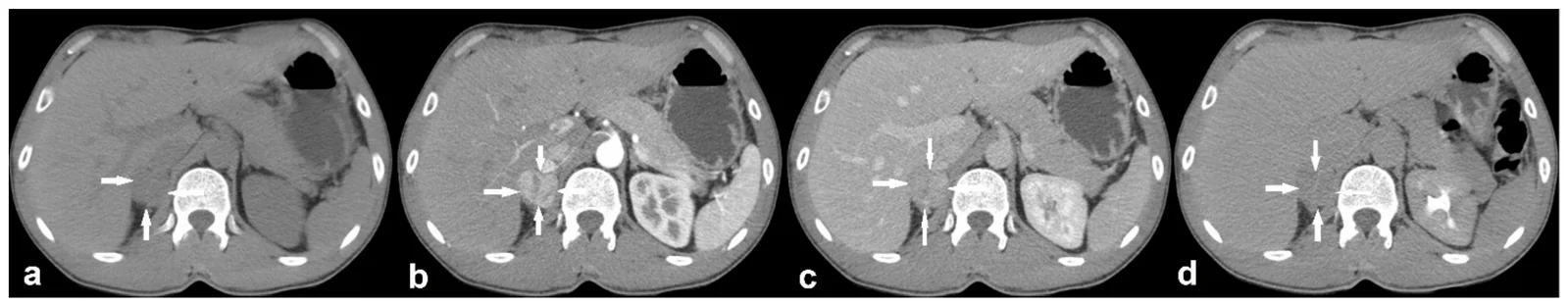
Axial CT images: (**a**) without contrast, (**b**) arterial phase, (**c**) venous phase, (**d**) late phase. The lesion showed a progressive increase in solid components, reduced cystic degeneration, and significant arterial-phase contrast enhancement, indicating a hypervascular adrenal tumor (arrows).

Preoperative CT scan performed in August 2025 demonstrated a persistent right adrenal lesion measuring approximately 3.0 × 2.8 cm. Compared with previous imaging, the lesion showed a progressive increase in its solid component, a reduction in cystic degeneration, and marked arterial-phase contrast enhancement, findings consistent with a hypervascular adrenal neoplasm (Figure 5).

**Figure 6:**
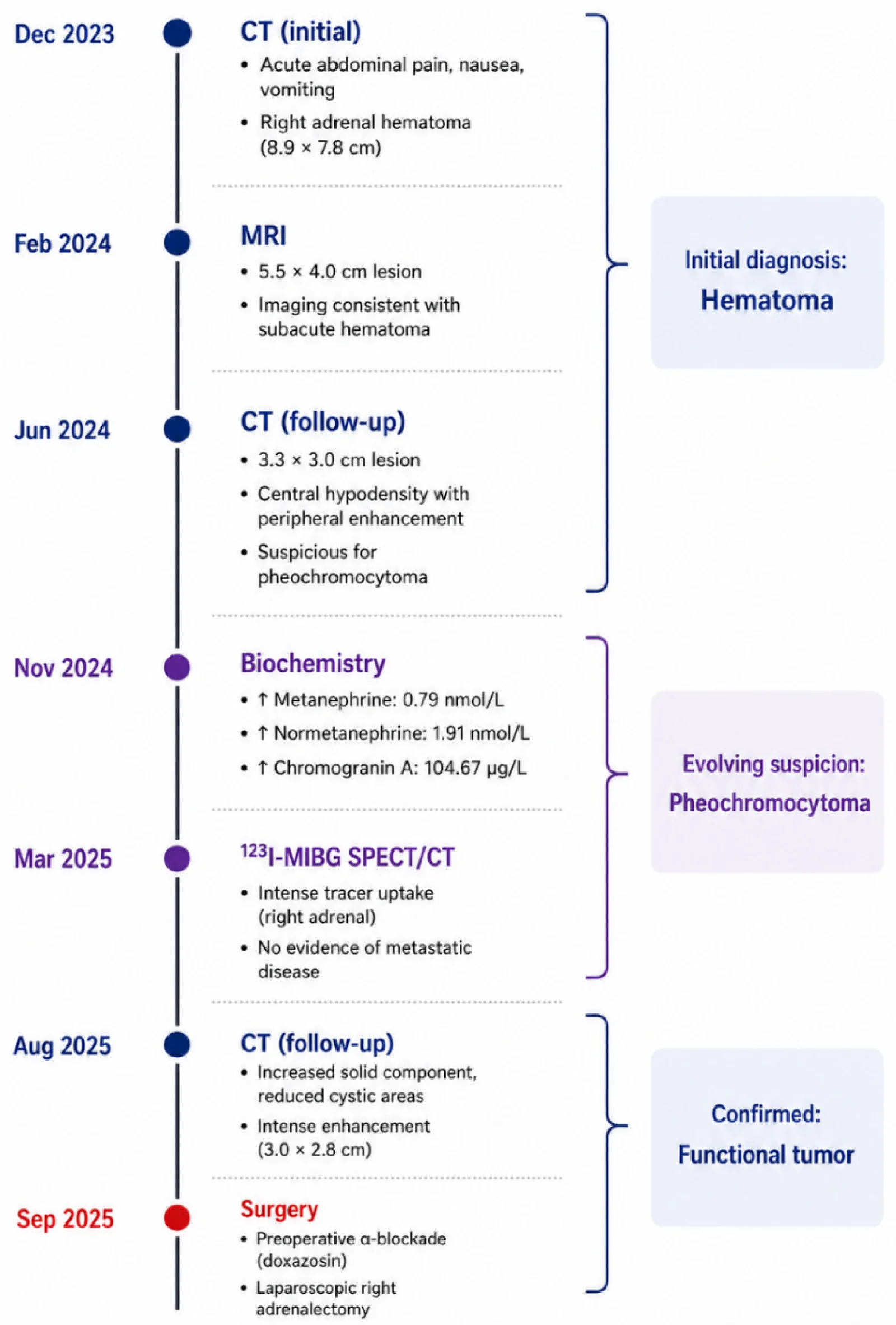
Timeline of the diagnostic course showing the evolution from an extensive adrenal hemorrhage obscuring the underlying lesion to the subsequent identification of pheochromocytoma.

Following multidisciplinary team discussion, a decision was made to proceed with surgical treatment, and the patient was scheduled for right adrenalectomy. After appropriate preoperative preparation with alpha-adrenergic blockade (doxazosin), the patient underwent laparoscopic surgery in September 2025. The procedure was completed without complications, and the postoperative course was uneventful. The patient was monitored for postoperative hypotension; however, no episodes occurred. Figure 6 presents the whole investigation timeline.

In February 2026, approximately five months after adrenalectomy, postoperative biochemical assessment showed normalization of plasma metanephrine (0.12 nmol/L), normetanephrine (0.30 nmol/L), and chromogranin A (40.93 μg/L), consistent with biochemical remission. The patient remained clinically well and asymptomatic and was normotensive, with a blood pressure of 118/77 mmHg and a heart rate of 73 bpm. Follow-up imaging had not yet been performed, as it was scheduled for one year after adrenalectomy.

Histopathological examination confirmed a pheochromocytoma of the right adrenal gland, with a predominantly solid and trabecular architecture and only a sparse Zellballen pattern; tumor cellularity was 150–250 cells per high-power field. No comedo-type necrosis or definite vascular invasion was identified. Histopathological examination demonstrated focal invasion of the adrenal capsule without extension into the periadrenal soft tissue; resection margins were clear (R0). Immunohistochemistry was positive for chromogranin A, and the Ki-67 proliferation index was approximately 1–3%. According to the Grading System for Adrenal Pheochromocytoma and Paraganglioma (GAPP), the tumor scored 4 points (moderately differentiated) and had a pathological stage of pT1NxMx. On gross examination, the tumor measured 3.1 × 2.5 × 3.0 cm within the resected adrenal gland (5.6 × 4.5 × 3.6 cm). No evidence of metastatic disease was identified. The patient was subsequently referred for endocrinological follow-up and genetic evaluation. Next-generation sequencing (Illumina NextSeq platform, OncoClever-GeneSGKit^®^ hereditary cancer gene panel; 12 genes analyzed: FH, MAX, MEN1, NF1, RET, SDHA, SDHAF2, SDHB, SDHC, SDHD, TMEM127, VHL) did not identify pathogenic variants associated with hereditary pheochromocytoma or paraganglioma syndromes.

## 3. Discussion

Hemorrhage in the adrenal region or retroperitoneal space presents a diagnostically challenging clinical scenario. Acute retroperitoneal bleeding may obscure the anatomy of the adrenal gland, complicating the differentiation between primary adrenal pathology and secondary hemorrhage during initial imaging assessments [4,7,8]. Consequently, the underlying etiology of the hemorrhage may remain unrecognized during the acute phase, and a definitive diagnosis is frequently established only through follow-up imaging after partial hematoma resolution [4,5,9]. This diagnostic delay was also observed in the present case, in which the initial imaging findings were interpreted as a retroperitoneal hematoma, and the adrenal gland was not visualized. Spontaneous hemorrhage was the initial manifestation of the tumor and resulted in acute symptoms that prompted urgent medical evaluation.

The pathophysiological mechanisms underlying hemorrhage in pheochromocytoma are not fully understood. Several mechanisms have been proposed, including increased tumor vascularity, fragile intratumoral vessels, ischemic necrosis, increased intracapsular pressure, and hemodynamic fluctuations related to catecholamine secretion [5,10,11]. These factors may result in intratumoral bleeding or hemorrhage extending into the retroperitoneal space, further complicating the diagnostic evaluation. Our patient remained normotensive despite harboring a catecholamine-secreting tumor. However, hypertension is considered a hallmark feature of pheochromocytoma; approximately 5–15% of patients are reported to be normotensive, making diagnosis particularly challenging in the absence of classic symptoms [12].

Imaging plays a central role in the evaluation of suspected adrenal or retroperitoneal hemorrhage. CT is usually the first imaging modality in patients presenting with acute abdominal symptoms, as it allows rapid detection of retroperitoneal bleeding and associated pathology [4,7,13]. Several studies have reported that the underlying adrenal pathology often becomes evident only on follow-up imaging after partial resorption of the hematoma [4,5,9]. In our case, this radiological evolution was clearly demonstrated. The initial hemorrhagic lesion measured approximately 8.9 × 7.8 cm and obscured the underlying adrenal pathology. Subsequent imaging showed a gradual reduction in the hematoma and progressive visualization of a persistent adrenal mass, eventually raising suspicion for pheochromocytoma. The interval between the initial hemorrhage and definitive surgery was approximately 21 months, from December 2023 to September 2025. During this period, the patient had multiple biochemical tests in November 2024, January 2025, and September 2025. These tests showed increasing plasma levels of normetanephrine (from 1.91 to 3.83 nmol/L) and chromogranin A (up to 133.42 μg/L). This prolonged diagnostic course reflected both the initial need for radiological follow-up as the extensive hematoma obscured the underlying adrenal lesion and the sequential nature of the subsequent diagnostic work-up. The relatively long intervals between these steps partly reflected scheduling and the availability of healthcare services in routine clinical practice.

MRI may provide additional diagnostic information by demonstrating heterogeneous signal intensity, cystic or hemorrhagic degeneration, and residual solid components suggestive of an underlying adrenal tumor [7,13,14]. In the present case, however, MRI findings remained consistent with a subacute hematoma and did not by themselves reveal the underlying tumor. Similar diagnostic delays have been described in the literature, particularly in patients presenting with spontaneous adrenal hemorrhage [5,15].

A key diagnostic step in such situations is follow-up cross-sectional imaging after hematoma resolution, which allows detection of a persistent adrenal lesion and differentiation between benign adrenal hematoma and hemorrhagic adrenal tumor.

Biochemical testing and functional imaging, including MIBG scintigraphy, are essential when a functional adrenal tumor is suspected [2,15,16,17,18,19]. Given the recognized hereditary background of pheochromocytoma and paraganglioma syndromes, the patient was referred for genetic evaluation. Next-generation sequencing did not identify pathogenic variants in the genes analyzed for these syndromes [20]. In the present case, the combination of elevated metanephrines and positive MIBG scintigraphy established the diagnosis before surgery. Notably, the patient remained normotensive throughout the diagnostic process, highlighting the importance of biochemical evaluation even in the absence of the classic clinical presentation of pheochromocytoma.

Recognition of pheochromocytoma before surgical intervention is critically important because manipulation of an undiagnosed catecholamine-secreting tumor may result in severe intraoperative hypertension, arrhythmias, or cardiovascular collapse [11,13,14]. Adequate preoperative preparation with alpha-adrenergic blockade remains a cornerstone of management and significantly reduces perioperative complications [6,21]. In our case, preoperative preparation with doxazosin was completed, and laparoscopic adrenalectomy was performed without intraoperative or postoperative hemodynamic complications. Once medical stabilization has been achieved, adrenalectomy is the definitive treatment and is generally associated with favorable outcomes when performed after appropriate endocrine preparation and multidisciplinary evaluation [9,21]. In cases of hemorrhagic pheochromocytoma, prior bleeding and surrounding hematoma may distort normal adrenal anatomy and obscure tissue planes, which can complicate surgical dissection and require careful intraoperative technique and hemodynamic monitoring [9,12,21].

Long-term follow-up is advised for all pheochromocytoma patients because each tumor has metastatic potential, with recurrence or metastasis possibly occurring years after initial treatment. Patients with adverse histopathological features (e.g., higher GAPP score or elevated Ki-67 index) should be monitored more closely for early detection of recurrence or metastasis [9,14]. The prognosis after complete resection is generally good, but only tumors with confirmed metastases are classified as malignant. Even tumors with favorable histology, such as this case with a moderate GAPP grade and low Ki-67 index, have a lower risk of recurrence or metastasis, mostly involving lymph nodes, bone, liver, or lungs. Recurrence rates vary, emphasizing the need for long-term surveillance, even after complete tumor removal [9,14].

This case emphasizes the importance of considering pheochromocytoma in the differential diagnosis of spontaneous adrenal or retroperitoneal hemorrhage, even in patients without typical clinical manifestations such as sustained hypertension or imaging evidence of an adrenal mass. Beyond the absence of classic hypertensive symptoms, the principal educational value of this case lies in its well-documented radiological evolution: serial imaging captured the transition from an extensive hematoma that initially obscured the adrenal gland to a persistent, progressively enhancing adrenal mass, ultimately identified as a hypervascular tumor—a sequence that prompted the biochemical and functional work-up which, together with only modestly elevated plasma metanephrines requiring cautious interpretation, ultimately established the diagnosis. Early consideration of pheochromocytoma, together with appropriate biochemical evaluation, functional imaging, and close radiological follow-up, is essential to ensure safe surgical management and optimal patient outcomes [3,14].

## 4. Conclusions

Spontaneous adrenal hemorrhage can be the first sign of pheochromocytoma and should not be dismissed as an isolated radiologic finding without further endocrine testing. The radiologic evolution from an extensive hematoma to a persistent, progressively enhancing adrenal mass, as illustrated in this case, may be the earliest clue to an underlying tumor and underscores the value of follow-up imaging after hematoma resolution. Because acute bleeding may obscure the tumor and mimic other causes of acute abdomen, diagnosis can be delayed unless pheochromocytoma is actively considered. In such cases, a structured diagnostic plan is crucial for identifying the cause and ensuring safe treatment. Although hemorrhagic pheochromocytoma is rare, it is a serious condition, and missing it can lead to preventable complications or death.

## Data Availability

The data supporting the findings of this study are not publicly available due to privacy and ethical considerations.

## Abbreviations

The following abbreviations are used in this manuscript:

CT	Computed tomography
^123^I-MIBG	Iodine-123 metaiodobenzylguanidine
GAPP	Grading System for Adrenal Pheochromocytoma and Paraganglioma
MRI	Magnetic resonance imaging
SPECT/CT	Single-photon emission computed tomography
